# A new service model for the treatment of severe anorexia nervosa in the community: the Anorexia Nervosa Intensive Treatment Team

**DOI:** 10.1192/pb.bp.113.044818

**Published:** 2014-10

**Authors:** Calum Munro, Victoria Thomson, Jean Corr, Louise Randell, Jennie E. Davies, Claire Gittoes, Vicky Honeyman, Chris P. Freeman

**Affiliations:** 1 Anorexia Nervosa Intensive Treatment Team (ANITT), NHS Lothian, Edinburgh, UK; 2 Department of Psychiatry, University of Edinburgh, UK; 3 Queen Margaret University, Edinburgh, UK

## Abstract

**Aims and method** A community intensive treatment service for severe anorexia nervosa is described. The service is multidisciplinary but driven by a focus on psychological formulation. Psychological and dietetic interventions are grounded in a process of active risk management. Evaluations of safety, cost and acceptability of the service are described.

**Results** Patients are highly satisfied with their care. A relatively low mortality rate for such a high-risk population was observed. In-patient bed use and costs were substantially reduced.

**Clinical implications** There is a case for greater use of intensive community care for patients with severe anorexia nervosa, as it can be acceptable to patients, relatively safe and cost less than admission.

There is limited evidence for the efficacy of particular service models or specific treatments for anorexia nervosa.^1-4^ The dearth of evidence is greatest for the subgroup of patients with severe anorexia nervosa. In-patient care for such patients is expensive^5,6^ without evidence of greater efficacy, yet care for severe eating disorders remains predominantly in hospitals. In-patient care will always be necessary for some individuals, but questions about when it is necessary and for whom it is most effective are rarely addressed. In-patient services reporting favourable outcomes often exclude from their data drop-outs or more resistant patients.^7,8^ Local experience over 25 years suggested that many patients failed to make a sustainable recovery with in-patient admissions and some deteriorated. This prompted consideration of an alternative model of care.

In the development and testing of a community-based service model for the treatment of patients with severe anorexia nervosa we have sought to answer a number of questions. Can intensive community treatment avoid or minimise the use of in-patient care? Can treatment for severe anorexia nervosa be delivered safely in the community? Is it acceptable to patients? Is it cost-effective? The aim of this paper is to describe the service model we have developed and to present preliminary evidence that begins to answer these questions.

## Method

### A tiered matched-care service model

The Anorexia Nervosa Intensive Treatment Team (ANITT) service is one component of a comprehensive tiered matched-care service model for eating disorders in the Lothian region. The region has a population of approximately 800 000, with a high proportion of students in higher education (approximately 70 000). Eating disorder cases are matched to the appropriate tier in relation to the severity of their presentation. Tier 1 involves guided self-help with either bibliotherapy or internet-based cognitive-behavioural therapy. Tier 2 is out-patient treatment including group and individual therapy, dietetic and psychiatric treatment. The ANITT service sits at the third tier. Tier 4 is specialist in-patient care. For an intensive service to function for the minority of patients with severe anorexia nervosa (about 30%), it is vital that less intensive out-patient treatment (Tier 2) and specialist in-patient care (Tier 4) are available and sufficiently resourced.

### ANITT staffing, capacity and organisation

The multidisciplinary team all work within a psychological framework, focusing on engagement. The team is composed of a consultant psychiatrist in psychotherapy (1.0 whole time equivalent (WTE)), a consultant clinical psychologist (0.8 WTE), two clinical psychologists (1.6 WTE), one clinical associate in applied psychology (0.8 WTE), two dieticians (1.8 WTE), one nurse (1.0 WTE), three assistant psychologists (3.0 WTE) and an administrator (0.7 WTE). The service is supported by an annual budget of £549 000, with capacity for 35 patients.

The service operates from a psychiatric hospital site, with open-plan office space facilitating communication. An extensive supervision structure is in place. The consultant psychiatrist and the consultant clinical psychologist lead the team with the support of a management group. There is significant travel to home visits across the Lothian region, an area of 4732 km^2^. We also use out-patient clinic space at the Tier 2 service. Over half of our clinical contacts are at home or in the community.

### Treatment pathway

**Fig 1:**
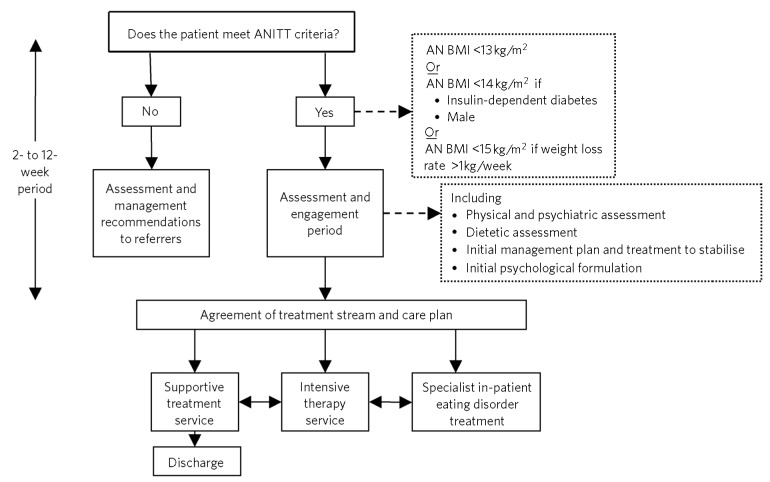
Anorexia Nervosa Intensive Treatment Team (ANITT) pathway. AN, anorexia nervosa; BMI, body mass index.

The core criteria we use for entry to the team are a body mass index (BMI) <13 kg/m^2^, or <15 kg/m^2^ and losing weight >1 kg per week. Patients initially enter a period of assessment, engagement and stabilisation. Assuming the patient is safe enough for community treatment, a decision is then reached about the appropriate treatment stream (Fig. 1).

### Assessment, engagement and stabilisation period

This is a physical, dietetic and psychological assessment process carried out over 2-12 weeks. Safety is continuously assessed. The initial focus of all team members is on achieving stability at a ‘safe enough’ weight. This is primarily achieved through dietetic and meal support work. The detailed dietetic assessment allows the fine tuning of nutritional intake and energy use around changes that the patient is willing and able to make. The close monitoring of physical risk indicators over time allows for a more sophisticated judgement of risk rather than simply ‘treating the BMI’. The therapist’s primary focus is on establishing a trusting relationship. The therapist establishes a preliminary developmental and maintenance formulation, to engage the patient and inform treatment planning. This process allows for a testing of the patient’s ability and willingness to engage in intensive therapy.

### Intensive therapy service

An 18-month course of twice-weekly therapy is offered with 6-monthly progress reviews. The patient must accept regular medical risk monitoring. Dietetic involvement is determined by the patient’s willingness and ability to engage. Assistant psychologists deliver meal support and social support work. The intensity of contact varies from two to ten contacts a week (weekdays), depending on progress and the stage of treatment. A 6-month period of reduced-intensity treatment follows the 18-month intensive period, to manage the risk of dependency and promote confidence and self-efficacy. This is tailored according to the patient’s needs. The treatment options at 2 years include discharge if a good recovery has been achieved, a further period of 6-12 months of intensive treatment or a period of supportive treatment.

### Supportive treatment service

For patients who are unwilling or deemed currently unable to benefit from intensive therapy, a period of supportive treatment is offered. This treatment package is more flexible and varied. The building blocks are continued physical and psychological risk management and the maintenance of a supportive relationship with the team. The focus is on supportive interventions with the aim of improving quality of life. The key professional with whom a long-term relationship is maintained may come from any member of the multidisciplinary team. The benefits of maintaining a long-term relationship in order to manage risk and work on improvements in quality of life appear to outweigh the risks of dependency.

### Psychological treatment

All multidisciplinary work is driven by an individual psychological formulation for that patient. Our core conceptualisation of anorexia nervosa is to consider the control of eating and weight as coping strategies which give our patients relief from overwhelming feelings of vulnerability. We therefore do not seek to threaten this by removing their main coping strategies until they begin to establish other means of meeting their needs. We work around the control of eating and weight as long as a minimum level of physical safety can be maintained.

The formal psychotherapy work in the intensive therapy service uses the schema therapy mode model.^9-11^ Schema therapy theory contends that we all have universal core emotional needs such as the need for safety, acceptance and nurturance, and if these are not adequately met this leads to psychological ill health. In anorexia nervosa basic physical needs, such as nutrition and the need for rest, are also not met. We offer two sessions a week for an 18-month course of therapy. Contrary to accepted wisdom, we find the majority of our patients, once engaged in a sufficiently trusting therapeutic relationship, able to engage in therapy even when at low BMIs. For those unable or unwilling to engage in intensive psychological work, we offer supportive therapy within the supportive treatment service. The therapy is flexible and draws on models of specialist supportive clinical management.^12^ The framework of unmet core emotional and physical needs is used with the intention of improving functioning and increasing insight and awareness of the restrictions their illness imposes on our patients.

### Dietetic treatment

The focus is on supporting the patient to make their own decisions about nutritional change. This is achieved by maintaining a focus on the patient’s aims and readiness to change. The dietician needs to display an empathic understanding of the difficulties of change while mastering the art of negotiating realistic small changes. Helping the patient hold on to the ‘big picture’ rather than getting caught up in the obsessional minutiae is also key. The dietician works on making realistic aims, tackling barriers to dietary change and coordinating the support needed to achieve their aims. There is close work with the other members of the team to ensure treatment is safe and integrated with the psychological work.

### Risk management

Safe management of risk is essential in a patient group with a relatively high mortality rate.^13^ Our systems of risk appraisal and management are openly shared with our patients. Key to the effectiveness of these systems is the relationship with the patient. A trusting relationship fosters openness from the patient allowing for better judgement of risk.

#### Patient risk management

We have a system for defining a physical state reflecting acute risk in anorexia nervosa, to guide when in-patient care is necessary (online Appendix DS1). Many patients describe experiences of being told they are at high risk of dying, do not become seriously unwell and therefore lose trust in clinicians. We think it is important not to exaggerate the risks but to make risk assessment as transparent and objective as possible. We do so using a descriptive ‘traffic light’ system (online Appendix DS2), routinely sharing their risk status with the patient, so the need for admission never comes as a surprise and is based on shared objective markers of physical risk. Inevitably, this is a process of judgement alongside objective criteria. The risk monitoring is largely carried out by the specialist nurse and the consultant psychiatrist.

The process of psychological risk management needs less explanation as it is largely a standard process as in other areas of mental health. For consistency we also use a ‘traffic light’ system to categorise our patient’s psychological risk.

#### Staff risk management

Burnout is a much acknowledged problem for clinicians working with eating disorders.^14^ It is essential that the staff feel ‘safe enough’ and are able to manage the inevitable anxiety integral to working with such high-risk patients in the community. We manage the risk of staff burnout in a number of ways: retaining small case-loads; an extensive supervision structure; a monthly reflective staff group facilitated by an external psychotherapist; and a culture of open and active communication.

## Results

We carried out a survey of 46 current or recently discharged patients in December 2010, of whom 33 (72%) completed a patient satisfaction questionnaire. To capture satisfaction ratings across all aspects of ANITT care, we developed a 6-item questionnaire constructed of Likert scales (1, not satisfied at all; 5, extremely satisfied), with an open text section for additional comments. There were no significant differences between satisfaction ratings on any of the six aspects of ANITT care. The mean overall satisfaction rating for all completers was 4.0. Analysis of open text comments revealed three main themes.

1. Staff perceived as supportive, caring and genuine: ‘This is the first period [of treatment] I feel truly understood, and able to trust... SO much effort is made with me from everyone. I feel genuinely supported.’
2. Patients valued a holistic psychological approach based on emotional and physical needs and not just weight: ‘The fact that... medical/nutritional/practical aspects were very well integrated into my psychotherapy was helpful.’
3. Patients valued individualised care: ‘Treatment is individualised and personal.’

We can describe reliable data on engagement, use of in-patient treatment and service costs for the period 2009-2011. The service expanded staff numbers and capacity to the current level in late 2008. During 2009-2011 we admitted to a single in-patient unit, a situation which subsequently changed. Consequently, 2009-2011 was a period when significant internal and external service variables remained constant and therefore outcome data can be more clearly interpreted.

### Engagement

Evidence of engagement in treatment was gathered from the patient satisfaction survey, treatment drop-out and Mental Health Act 1983 use, for this notoriously hard to engage patient population. The high overall satisfaction rating and open text themes from our survey reflect a largely engaged patient population. Only two patients during 2009-2011 dropped out of the service and only five patients required detention under the Mental Health Act. One patient was detained using a community treatment order at a BMI of 11 kg/m^2^ to ensure engagement in medical monitoring and dietetic treatment. Three patients were detained due to starvation-related risks. One patient was detained due to suicide risk following a significant overdose. All four patients detained for admission had comorbid personality disorder diagnoses and required prolonged periods of in-patient care.

### Service costs

**Fig 2:**
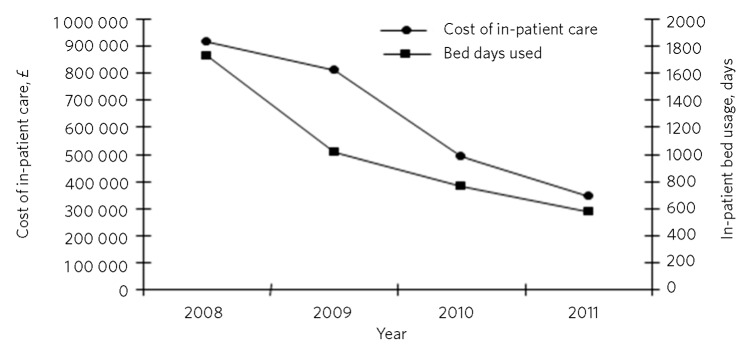
Anorexia Nervosa Intensive Treatment Team in-patient usage and costs 2008-2011.

Service cost is an important outcome for a service providing intensive treatment for a severely ill patient group usually treated in an in-patient setting. In the year 2008 prior to the expansion of the team, the service cost £370 000 and in-patient admissions cost £918 208, a total annual cost of care for the severe anorexia nervosa population of £1 288 208. During 2009-2011, in-patient care reduced due to increased capacity to deliver intensive treatment in the community (Fig. 2). In 2011, in-patient care costs were £347 552 and the ANITT service cost was £549 000, a total annual cost of care of £896 552. A total saving of £391 656 in 2011 compared with the index year of 2008 prior to the service expansion.

### Patient safety

Patient safety is the most important outcome. A significant mortality rate would be expected in a service selecting patients with severe anorexia nervosa. Mortality data can be most meaningfully described for the full 8-year period of the service’s existence. Of all 101 patients treated in the service over this 8-year period, 4 have died, giving a crude mortality rate of 4%. All four had a duration of illness of over 10 years. One patient died of starvation-related causes while an in-patient; one died of an overdose after weight restoration; the third patient died of post-operative septicaemia following acute bowel obstruction having dropped out of ANITT care; and the fourth patient died of pneumonia and heart failure with comorbid insulin-dependent diabetes, in the context of a starvation state.

## Discussion

There is very little reliable evidence for the efficacy of in-patient care for adults with anorexia nervosa. A recent randomised controlled trial has shown the potential for managing patients with chronic anorexia nervosa in the community^15^ and one other community-based treatment programme has described promising preliminary outcomes.^16^ We describe preliminary evidence that patients specifically with severe anorexia nervosa can be treated safely in the community, that patients value a community-based service and that admissions to in-patient care and costs can be substantially reduced. We aim to publish data on quality of life and symptomatic outcomes for a cohort of ANITT patients in the near future.

Our crude mortality rate of 4.0% (over an 8-year period) for patients with severe illness compares well with crude mortality rates of 3.4-4.4% reported for services treating anorexia nervosa populations across the severity spectrum.^17-19^ Two studies report mortality data for populations of similar severity to ours. Tanaka *et al*^20^ report a crude mortality rate of 11.5% over an 8-year follow-up period. A 14-year follow-up of a Swedish cohort with severe eating disorder analysed standardised mortality ratios (SMRs) according to lowest BMI around admission. The authors found that SMRs were not substantially increased across the BMI spectrum from 11.5 to 17.5 kg/m^2^, but increased significantly at the lowest BMIs <11.5 kg/m^2^.^21^ This evidence is consistent with our relatively low mortality rate despite regularly managing patients in the community with BMIs in the range 11.5-13.5 kg/m^2^. The Swedish data and our experience would suggest that managed and stabilised low weight may allow for adequate physiological adaptation, substantially reducing the risk of serious medical complications or death. We are firmly of the belief that developing trusting, long-term relationships is key to risk management. The transparent sharing of the risk assessment with patients leads to fewer surprises and less opposition from patients when admission is necessary.

Our financial data provide a clear example for hospital managers and service commissioners of ‘invest to save’, with adequate investment in a community care service resulting in substantial savings on in-patient care costs. Given the expense of in-patient care and the lack of evidence for its efficacy,^2^ unless patients are ready to change,^22^ we argue that in-patient care should be used sparingly for brief admissions wherever possible.^23^ We admit for three reasons: for stabilisation of acute medical (or psychiatric) risk; for initiation of weight gain for those motivated to do so who have been unable to gain weight at home; for treatment resistance in patients with persistent high risk. Our experience is that treating patients at home produces a wealth of contextual information that enriches the dietetic treatment and psychotherapy, in an environment where the patient feels safer. We believe this leads to more sustainable change, more often, than in an in-patient ward.

We are also aware of many professionals’ concerns about starvation-related cognitive deficits preventing engagement in psychological treatments, often used to justify the necessity of in-patient re-feeding. A small number of studies have explored cognitive, perceptual and socioemotional deficits in patients with anorexia nervosa in a starved state and in patients who have recovered.^24-29^ A mixed picture of whether these deficits resolve on recovery emerges. However, of the two studies that specifically tested patients who had recently restored weight after in-patient treatment,^24,25^ neither showed an association between weight gain and improved deficits. This may suggest that starvation is not the central cause of these deficits and that what evidence exists of improved cognitive, perceptual or socioemotional deficits in recovered patients may be more to do with the psychological than the physical recovery.

In our opinion therefore, there is insufficient evidence of a causal relationship between starvation, cognitive abnormalities and a failure to engage in psychotherapy and therefore this in itself is not a rationale for admission and re-feeding. An alternative psychological hypothesis to explain what is often labelled as starvation-related ‘cognitive deficit’ is that this is the emotional detachment and defensiveness of an individual feeling emotionally vulnerable within an insufficiently trusting therapeutic relationship. Our clinical experience is that emotionally engaged, trusting therapeutic relationships can be established with the majority of patients with severe anorexia nervosa, even at very low BMIs. This takes persistence and patience. Re-feeding resistant patients within an in-patient programme, however sensitively achieved, necessitates the removal of their core coping strategy and their sense of autonomy and control. For many patients, this results in fear, resistance, defensiveness and a loss of trust in professionals.

We therefore argue that in-patient care is overused because of fears about physical safety rather than objective evidence of acute risk and because of beliefs about starvation-related cognitive deficits preventing engagement in therapy. There is a case for intensive community care for patients with severe anorexia nervosa as it can be acceptable to patients, relatively safe and cost less than admission.
